# Greater political commitment needed to eliminate malaria

**DOI:** 10.1186/s40249-019-0542-8

**Published:** 2019-04-28

**Authors:** Minghui Ren

**Affiliations:** Universal Health Coverage, Communicable and Noncommunicable Diseases, World Health Organization, Geneva, Switzerland

**Keywords:** Malaria, Epidemic, Sustainable development goals, Poverty, Africa, Primary health care, Vector-borne diseases, Health funding, World malaria day

## Abstract

Malaria-related mortality has a very high association with poverty rates, and the disease is most prevalent in low- and middle-income countries. To achieve the malaria-specific targets of the Sustainable Development Goals, malaria-endemic countries and development partners need to take concerted action to reduce malaria cases and deaths. Reaching all affected communities with malaria interventions requires strong political commitment and a significant expansion of international and domestic financial resources. World Malaria Day 2019 is an opportunity to review progress and challenges in this field.

**Electronic supplementary material:**

The online version of this article (10.1186/s40249-019-0542-8) contains supplementary material, which is available to authorized users.

## Multilingual abstracts

Please see Additional file 1 for translations of the abstract into the five official working languages of the United Nations.

## Background

Since 2000, significant progress has been achieved in reducing the number of malaria cases and deaths around the world. According to the latest available estimates, between 2010 and 2017, the number of malaria cases was cut from an estimated 239 million to 219 million despite an increase in the population at risk [1]. In 2017, most malaria cases were in sub-Saharan Africa (92%), followed by south-east Asia (5%). Fifteen countries in sub-Saharan Africa and India carried almost 80% of the global malaria burden, with the highest-burden countries being Nigeria (25% of all cases worldwide) and the Democratic Republic of the Congo (11% of all cases). Malaria-related mortality has been reduced from an estimated 607 000 in 2010 to 435 000 in 2017 [1] Tables 1 and 2.

**Table 1:** Estimated malaria cases, 2010–2017. Estimated cases are shown with 95% upper and lower *CI*

	Number of cases (000)							
	2010	2011	2012	2013	2014	2015	2016	2017
Lower 95% *CI*	218 600	210 500	206 700	200 500	199 600	198 700	200 400	202 800
Estimated total	238 800	229 100	226 400	221 000	217 100	214 200	216 600	219 000
Upper 95% *CI*	285 400	273 200	271 600	266 200	259 300	257 200	259 000	262 000
Estimated *Plasmodium vivax*								
Lower 95% *CI*	11 440	10 390	9190	7040	6040	5530	5960	5720
Estimated total	16 440	14 940	13 300	10 230	8720	7950	8250	7510
Upper 95% *CI*	24 560	23 970	22 050	17 240	12 730	11 410	11 300	9900

*CI* Confidence interval, *WHO* World Health Organization

Source: WHO estimates. https://www.who.int/malaria/publications/world-malaria-report-2018/report/en/

**Table 2:** Estimated malaria cases by WHO region, 2017. Estimated cases are shown with 95% upper and lower *CI*

	Number of cases (000)					
	African	Americas	Eastern Mediterranean	South-East Asia	Western Pacific	World
Lower 95% *CI*	184 500	880	3630	8560	1395	202 800
Estimated total	200 500	976	4410	11 290	1857	219 000
Upper 95% *CI*	243 600	1128	5560	14 840	2399	262 000
Estimated *Plasmodium vivax*						
Lower 95% *CI*	19	648	1162	2881	330	5720
Estimated total	701	723	1366	4200	523	7510
Upper 95% *CI*	2197	843	1773	5900	774	9900
Proportion of *P. vivax* cases	0.3%	74.1%	31.0%	37.2%	28.1%	3.4%

*CI* Confidence interval, *WHO* World Health Organization

Source: WHO estimates. https://www.who.int/malaria/publications/world-malaria-report-2018/report/en/

However, cases and deaths are not coming down fast enough to meet the milestones and goals set by countries in the World Health Organization (WHO) *Global Technical Strategy for Malaria 2016–2030*, which provides a technical roadmap for the achievement of the relevant Sustainable Development Goals (SDGs) target. What is even more disconcerting, the rate of reduction in malaria incidence and mortality has slowed since 2015, with spikes in cases being reported by many countries around the world [1]. Between 2016 and 2017, the number of cases increased in all 10 countries with the highest malaria burden in Africa [1]. This is a major cause for concern and urgent action is needed to put the response back on track (Table 3).

**Table 3:** Estimated number of malaria deaths by WHO region, 2010–2017

	Number of deaths							
	2010	2011	2012	2013	2014	2015	2016	2017
African	555 000	517 000	489 000	467 000	446 000	432 000	413 000	403 000
Americas	480	450	400	400	300	320	460	630
Eastern Mediterranean	8070	7280	7340	6750	8520	8660	8160	8300
European	0	0	0	0	0	0	0	0
South-East Asia	39 800	32 800	28 400	21 800	24 100	25 200	25 600	19 700
Western Pacific	3770	3340	3850	4600	4420	2860	3510	3620
World	607 000	561 000	529 000	500 000	483 000	469 000	451 000	435 000
World (children aged under 5 years)	444 600	405 000	371 000	344 000	322 000	302 000	283 000	266 000

*WHO* World Health Organization

Source: WHO estimates. https://www.who.int/malaria/publications/world-malaria-report-2018/report/en/

## Main text

On 25 April 2019, countries around the world will mark World Malaria Day under the theme “Zero malaria starts with me”. This offers an opportunity to celebrate successes and review ongoing challenges in the fight against this preventable and treatable parasitic disease.

Despite significant gains over the last 18 years, malaria continues to pose a major global health threat. The disease remains endemic in 87 countries and takes an estimated 435 000 lives each year. Most of these deaths occur in the world’s poorest countries. While progress is on track on the majority of health-related SDG indicators for which information is available [2], global malaria efforts have substantially slowed in the past few years. WHO has issued multiple alerts regarding the slowdown and, in 2019, the organization listed malaria as an area where “progress has stalled or trends are in the wrong direction” in the broader context of health-related SDGs [2].

While comprehensive analysis is not available on the complex reasons for this slowdown, it is estimated to have been triggered by a combination of insufficient investments in malaria programmes (leaving major gaps in intervention coverage) as well as weak malaria surveillance systems, and an increasing number of complex emergencies. In Venezuela alone the long-standing political turbulence has put the health system under stress and triggered intense migration to malaria-endemic areas, which has led to a rise in malaria cases. Meanwhile, the ongoing conflict and instability in parts of the Democratic Republic of the Congo, Nigeria and other countries, is also thought to have contributed to a weakening of programme coverage.

### Malaria and poverty

It has been well established that malaria is both a cause and a consequence of poverty and social inequality. The disease burden is highest in Africa’s poorest countries and in India, which have significant rates of extreme poverty and income inequality. Within countries, the poorest segments of society – particularly children, women and other vulnerable groups including migrants, refugees and displaced populations – are most affected. The presence of malaria transmission affects both the health status of individuals and the economic potential and productivity of individuals and families. Numerous analyses have concluded that malaria transmission can have an overall negative impact on the economic growth of a country or region [3–5].

A recent WHO analysis on the association between the 40 leading causes of death and the concentration of deaths according to national income status found that malaria mortality has the *highest* association with national low-income status [2]. Another analysis found that malaria incidence is 75 times higher in low-income countries than in upper-middle income countries, while tuberculosis incidence is only 4 times higher, and new human immunodeficiency virus (HIV) infections are 2.8 times higher. Maternal deaths are 9 times higher in low-income countries than in upper-middle income countries [2].

### Need for increased funding

The close association between malaria and poverty calls for a significant expansion of the funding available for malaria programmes in affected countries. Each year, at least United States dollar (USD) 4.4 billion is needed globally to reach the targets of the WHO *Global Technical Strategy for Malaria*, with funding requirements expanding to at least USD 6.6 billion by the end of 2020. In 2017, only USD 3.1 billion was available – the same level of funding as in 2013 [1]. Despite commitments expressed in the Abuja Declaration (African Union, 2001) and the 2030 Agenda for Sustainable Development, most malaria-endemic countries have not increased their domestic funding for malaria control and elimination. Of the 41 high-burden countries, Cameroon and Mauritania have succeeded in more than doubling available funds for malaria programmes, and six others achieved a more than 20% increase [1] but around the world the global total of domestic funding spent on malaria has not changed since 2012 [1].

While increased national political commitment is urgently needed to make further progress, the continued engagement of the Global Fund to Fight AIDS, Tuberculosis and Malaria and donor governments is also critical. The Global Fund and bilateral donors (including the United States, United Kingdom, France, Germany, Canada, European Institutions, Japan and the Bill & Melinda Gates Foundation) currently provide an estimated two-thirds of all of the world’s malaria-related funding [1]. China is increasingly offering support through the South-to-South collaboration framework. The Global Fund’s replenishment conference in October 2019 will be an important opportunity for existing and new donors to pledge new funding to fill current gaps. The funding is needed to expand access to preventive measures, quality-assured diagnostics and treatment, strengthen surveillance systems and to continue integrating malaria programmes into primary health care and other health system platforms.

It has been well established that health spending on malaria control and other major infectious diseases generates a significant return on investment. According to a recent report, in the WHO African Region the full achievement of the SDG3.3 targets related to HIV/AIDS, tuberculosis, malaria and neglected tropical diseases would reduce estimated productivity losses caused by these infections to such a degree that the benefits would greatly surpass the costs of health interventions into controlling those diseases [6].

### Moving towards increased country impact

In order to encourage stronger political and financial commitment in the highest-burden countries, WHO and the RBM Partnership to End Malaria are advocating for a new approach. The “High burden high impact” approach is anchored by 4 pillars. It calls for (1) the translation of political commitments into financial resources and tangible actions that will save more lives; (2) a move away from a one-size-fits-all approach by a strengthened and more strategic use of data in order to deploy control tools for maximum impact; (3) improved and targeted global policies and strategies to help countries deliver the optimal mix of tools for their unique settings; and (4) a coordinated country response to ensure alignment of partners and engagement of sectors beyond health.

The development of new tools and strategies for the prevention, diagnosis and treatment of malaria is also critical to accelerating progress towards the 2030 goals. In 2018, WHO released a list of prioritized malaria-related health products [7], which included transmission blocking medicines, simplified therapies, novel vector control tools, and preventive and transmission blocking vaccines for both *Plasmodium falciparum* and *P. vivax* malaria. For medicines and vaccines, the malaria pipeline is already strong and the world’s first malaria vaccine, RTS,S/AS01, is expected to be launched this year in a landmark pilot programme in three African countries. However, in many other areas, significant investments are needed in research and development to strengthen the pipelines.

Taking a health systems perspective – i.e. strengthening malaria programmes through integration with community-based primary health care programmes or other vector-borne disease programmes, where relevant – can also help to create sustainable foundations for malaria programmes. This is increasingly important as a large subset of neglected tropical diseases affect many of the same communities as malaria but have different service delivery platforms in place. Also, other mosquito-borne diseases – such as dengue, chikungunya and Zika – are on the rise, and their control requires increasingly integrated approaches. Following a request by WHO Member States, WHO developed a new integrated global vector control strategy in 2017, the *Global Vector Control Response 2017–2030* [8], which provides guidance to countries on the integration and scale-up of relevant programmes. Tackling the social determinants of the malaria epidemic, such as poverty and social inequalities, will also strengthen national malaria responses.

### Successes in eliminating malaria

Despite the challenge that malaria poses around the world, many countries have succeeded in moving closer to elimination, or have eliminated the disease. Since 2000, WHO has certified 9 countries as malaria-free [9] and between 2010 and 2017, the number of countries with fewer than 10 000 reported cases increased from 37 to 46. Some high-burden countries have also managed to significantly cut their incidence rates – for example, India registered a 24% reduction in cases in 2017, compared to 2016 [1]. Other countries that reported considerable declines include Ethiopia, Pakistan and Rwanda.

Countries that have successfully reduced their malaria burdens have applied similar strategies: they invested heavily in the scale-up of prevention tools, expanded diagnostic testing and treatment, and significantly strengthened their malaria surveillance systems. On World Malaria Day this year, WHO joins the RBM Partnership to End Malaria, the African Union Commission and other partners in promoting the “Zero malaria starts with me” campaign that seeks to keep malaria high on the political agenda, mobilize additional resources and empower communities to improve their access to malaria prevention and care.

## Conclusions

Given its strong association with poverty, malaria is a litmus test for the achievement of the global ambition to ‘leave no one behind’. Robust and coordinated action is needed by all endemic countries, donors, the private sector, academia and research organizations to strengthen malaria responses and prevent the unbearable loss of life. Countries and partners should work together to embrace the current global momentum around universal health coverage and raise additional financial resources for malaria programmes. At the same time, countries are urged to move towards integrating malaria programmes into people-centred primary health care services in order to ensure long-term sustainability of malaria responses.

## Additional file

Additional file 1: Multilingual abstracts in the five official working languages of the United Nations. (PDF 468 kb)

## Abbreviations

AIDS: Acquired immune deficiency syndrome
HIV: Human immunodeficiency virus
SDGs: Sustainable Development Goals
USD: United States dollar
WHO: World Health Organization
