# Supplementary material for: Greater political commitment needed to eliminate malaria
Source: Infect Dis Poverty. 2019 Apr 28;8:28. doi: 10.1186/s40249-019-0542-8 (PMC6487040; doi:10.1186/s40249-019-0542-8)
Supplement: Supplementary file 1 — Multilingual abstracts in the five official working languages of the United Nations. (PDF 468 kb) [file 40249_2019_542_MOESM1_ESM.pdf]

Translation of the abstract into the five official working languages of the United Nations

يتطلب القضاء على الملاريا مزيدا من الالتزام السياسي

Ren Minghui

موجز

ترتبط الوفيات المرتبطة بالملاريا ارتباطا وثيقا بمعدلات الفقر ، وينتشر المرض بشكل كبير في البلدان المنخفضة الدخل والبلدان المتوسطة الدخل. لتحقيق الأهداف الخاصة بالملاريا في أهداف التنمية المستدامة ، تحتاج البلدان الموبوءة بالملاريا والشركاء في التنمية إلى اتخاذ إجراءات منسقة للحد من حالات الملاريا والوفيات الناجمة عنها. ويتطلب الوصول إلى جميع المجتمعات المتأثرة بتدخلات مكافحة الملاريا التزامًا سياسيًا قويًا واتساعا كبيرًا في الموارد المالية الدولية والمحلية. إن اليوم العالمي لمكافحة الملاريا لعام 2019 فرصة لاستعراض التقدم المحرز والتحديات في هذا المجال.

加强领导，消除疟疾

Ren Minghui

摘要

疟疾所致死亡率与贫困率密切相关，而且疟疾在中低收入国家最为普遍。为实现可持续发展目标中的疟疾相关指标，疟疾流行国家和发展伙伴需齐心协力，降低疟疾病例数和死亡人数。在所有受影响的社区开展疟疾干预措施，需要强有力的政治支持，以及国际社会和流行国家大幅增加资金支持力度。值此 2019 年世界疟疾日之际，本文分析了该领域的进展和挑战。

Grand engagament politique nécessaire à éliminer le paludisme

Ren Minghui

Résumé

La mortalité relative au paludisme s’associe étroitement avec le taux de pauvreté, et la maladie est la plus présente dans des pays de revenus faibles and moyens. Pour atteindre les objectifs spécifiques de paludisme au niveau des Buts de Développement Durable, les pays de paludisme endémique et les partenaires de développement doivent prendre action commune à réduire des cas et morts de paludisme. L’arrivée à toutes les communes affectées avec l’intervention de paludisme requiert un engagement politique puissant et une expansion significative des ressources financières à l’intérieur et l’extérieur du pays. La Journée de Paludisme mondiale 2019 est une opportunité à réfléchir aux progrès et aux défis dans ce domaine.

Необходима масштабная политическая приверженность для ликвидации малярии

Ren Minghui

Аннотация
Смертность от малярии крайне тесно связана с уровнем бедности. Эта болезнь наиболее распространена в странах с низким и средним уровнем дохода. Для достижения целей, связанных с малярией, которые входят в перечень Целей устойчивого развития, страны, страдающие от малярии, и партнеры по развитию должны предпринять согласованные действия для сокращения случаев заболевания малярией и смертности от нее. Чтобы реализовать меры по борьбе с малярией, охватив все пострадавшие общины, требуется всецелая политическая приверженность и значительное увеличение международных и внутренних финансовых ресурсов. Всемирный день борьбы с малярией в 2019 году - это возможность оценить достижения и проблемы в этой области.

Un mayor compromiso político es necesario para eliminar la malaria.

Ren Minghui

Resumen
Existe una asociación estrecha entre la tasa de pobreza y la mortalidad que se relaciona con la malaria, y la enfermedad es más amplia en los países de ingresos bajos y medios. Para lograr metas concretas de la malaria en los Objetivos de Desarrollo Sostenible, y con motivo de reducir los casos y las muertes por malaria, los países endémicos de la malaria y los socios de desarrollo deben tomar medidas consistentes. Requiere un fuerte compromiso polí tico y una expansión significativa de los recursos financieros nacional e internacional para llegar con intervenciones a todas las comunidades afectadas de malaria. El Día Mundial de la Malaria de 2019 constituye una oportunidad para reflexionar sobre el avance y los desafíos en este campo.
